# Supplementary material for: COVID-19 Vaccines: Fear of Side Effects among German Health Care Workers
Source: Vaccines (Basel). 2022 Apr 28;10(5):689. doi: 10.3390/vaccines10050689 (PMC9146316; doi:10.3390/vaccines10050689)
Supplement: Supplementary file 1 [file vaccines-10-00689-s001.zip › vaccines-1665360-supplementary.pdf]

COVID-19 vaccine: Fear of side effects among German health care workers

Supplemental Materials

Supplemental Table S1: Invitations and responses from German regions and federal states (taken from Holzmann-Littig et al, 2021 (1)

Federal state	Number of e-mail invitations (% of total)	Number of responses in the final dataset (% of total)	Number of inhabitants Dec. 31st, 2019 [Millions], (% of total)
**Northern Germany**			
Schleswig-Holstein	183 (4.7)	516 (11.5)	2.904 (3.5)
Mecklenburg-Western Pomerania	174 (4.4)	34 (0.8)	1.608 (1.9)
Hamburg	171 (4.3)	94 (2.1)	1.847 (2.2)
Lower Saxony	227 (5.8)	593 (13.2)	7.994 (9.6)
Bremen	152 (3.9)	29 (0.6)	0.681 (0.8)
Total Northern Germany	907 (23.1)	1266 (28.1)	15.034 (18.1)
**Eastern Germany**			
Brandenburg	195 (5.0)	81 (1.8)	2.522 (3.0)
Berlin	196 (5.0)	110 (2.4)	3.669 (4.4)
Saxony-Anhalt	174 (4.4)	61(1.4)	2.195 (2.6)
Saxony	212 (5.4)	81 (1.8)	4.072 (4.9)
Thuringia	167 (4.3)	47 (1.0)	2.133 (2.6)
Total Eastern Germany	944 (24.1)	380 (8.4)	14.591 (17.5)
**Western Germany**			
North Rhine-Westphalia	292(7.4)	643 (14.3)	17.947 (21.6)
Hesse	229 (5.8)	400 (8.9)	6.288 (7.6)
Rhineland-Palatinate	223 (5.7)	94 (2.1)	4.094 (4.9)
Saarland	150 (3.8)	31 (0.7)	0.987 (1.2)
Total Western Germany	894 (22.8)	1168 (26.0)	29.316 (35.2)
**Southern Germany**			
Bavaria	375 (9.6)	1380 (30.7)	13.125 (15.8)
Baden-Wuerttemberg	704 (17.9)	202 (4.5)	11.100 (13.3)
Total Southern Germany	1079 (27.5)	1582 (35.2)	24.225 (29.1)
**Nationwide**			
Non region related organizations:	100		
Germany total	3924		83.166
No answer to federal state question		104 (2.3)	
Datasets used		4500	

Supplemental Table S2. Original questions of the survey, translated from German, taken from Holzmann-Littig et al., 2021.(1)

**Section 1: Basic data / demographics**
**Sex**
female / male / no answer
**Age**
≤20 years / 21-30 years / 31 - 40 years / 41 - 50 years / 51 - 60 years / ≥61 years / no answer
**Profession**
Certified nurse / Non-examined nurse / Medical specialist (Medical technical assistants, surgical assistant etc.) / Resident physician / specialized physician / Consultant physician / Chief physician / administrative staff / Non-

physician staff in the rescue service / Trainee / student of human medicine / Student of dentistry / other – Free text / no answer
**Work Setting**
Maximum-care hospital; university hospital / Hospitals of other care levels<br><br>Medical practice; Medical care center / Rescue service / nursing home; retirement home / Outpatient nursing service / other – free text / no answer
**You indicated that you work in an inpatient setting. Do you work in an intensive care unit in this setting? If work setting 1 or 2 have been selected.**
yes / no
**I work with COVID-19 positive patients**
Never / under 50% of my working days / over 50% of my working days (but not every working day) / on each of my working days / no answer
**I work in the following federal district:**
Bavaria / Baden-Wuerttemberg / Berlin / Brandenburg / Bremen / Hamburg / Hesse / Mecklenburg-Western Pomerania / Lower Saxony / North Rhine-Westphalia / Rhineland-Palatinate / Saarland / Saxony / Saxony-Anhalt / Schleswig-Holstein / Thuringia / no answer
**Section 2: Vaccinations in general**
**Please rate the following statements about vaccinations in general:**
**I make sure to keep my vaccinations up to date.**
I do not agree at all / I rather do not agree / I neither agree nor disagree / I rather agree / I fully agree / no answer
**I receive flu (influenza) vaccinations regularly.**
I do not agree at all / I rather do not agree / I neither agree nor disagree / I rather agree / I fully agree / no answer
**I feel well informed about vaccines in general.**
I do not agree at all / I rather do not agree / I neither agree nor disagree / I rather agree / I fully agree / no answer
**I am generally afraid of adverse effects of vaccinations.**
I do not agree at all / I rather do not agree / I neither agree nor disagree / I rather agree / I fully agree / no answer
**I think vaccinations are generally effective.**
I do not agree at all / I rather do not agree / I neither agree nor disagree / I rather agree / I fully agree / no answer
**I trust vaccines in general.**
I do not agree at all / I rather do not agree / I neither agree nor disagree / I rather agree / I fully agree / no answer
**I believe that the pharmaceutical industry puts profit motives over the safety of vaccines.**
I do not agree at all / I rather do not agree / I neither agree nor disagree / I rather agree / I fully agree / no answer
**I trust the regulatory authorities of vaccines in Germany in general.**
I do not agree at all / I rather do not agree / I neither agree nor disagree / I rather agree / I fully agree / no answer
**I already had a serious vaccine adverse effect that required medical treatment.**
yes / no / no answer, if yes -> free text

**Section 3 Vaccinations against COVID-19**
**I have already received one or two vaccinations against COVID-19 or already have a vaccination appointment.**
yes / no / no answer
**In my personal environment (family, friends, colleagues) there are people who have already received one (or two) vaccinations.**
yes / no / I do not know / no answer
**I want to get vaccinated against COVID-19. (Questions opens only if participant indicates not to have been vaccinated yet / if participant clicked "no answer".)**
yes / no / undecided / no answer
**I do not want to get vaccinated until I see that a lot of people who have been vaccinated have tolerated it. (Question only opens, if "undecided" or "no answer" have been indicated.**
I do not agree at all / I rather do not agree / I neither agree nor disagree / I rather agree / I fully agree / no answer
**In my personal environment (family, friends, colleagues) there were people suffering from COVID-19.**
yes / no / I do not know / no answer
**You have indicated that there were COVID-19 patients in your personal environment. Please answer the following questions: (Question only opens, if "yes" has been indicated.)**
**One / more persons of the above have been hospitalized in connection with COVID-19 disease**
yes / no / I do not know / no answer
**One / more persons of the above have been in an intensive care unit in connection with the COVID-19 disease**
yes / no / I do not know / no answer
**One / more persons of the above have died in connection with COVID-19 disease**
yes / no / I do not know / no answer
**In my personal environment (family, friends, colleagues) there are people for whom a COVID-19 disease would probably be severe.**
yes / no / I do not know / no answer
**Please answer the following questions on COVID-19 / vaccinations against COVID-19:**
**In case of COVID-19 disease, I fear a severe course of the disease for myself**
I do not agree at all / I rather do not agree / I neither agree nor disagree / I rather agree / I fully agree / no answer
**I am afraid of getting infected with COVID-19 in my professional environment.**
I do not agree at all / I rather do not agree / I neither agree nor disagree / I rather agree / I fully agree / no answer
**I am afraid of becoming infected with COVID-19 in a private environment.**
I do not agree at all / I rather do not agree / I neither agree nor disagree / I rather agree / I fully agree / no answer
**I feel well informed about COVID-19 vaccines.**
I do not agree at all / I rather do not agree / I neither agree nor disagree / I rather agree / I fully agree / no answer
**I am afraid of short-term adverse effects of COVID-19 vaccines.**
I do not agree at all / I rather do not agree / I neither agree nor disagree / I rather agree / I fully agree / no answer

**I am afraid of long-term adverse effects from COVID-19 vaccines.**
I do not agree at all / I rather do not agree / I neither agree nor disagree / I rather agree / I fully agree / no answer
**I believe the COVID-19 vaccines are effective.**
I do not agree at all / I rather do not agree / I neither agree nor disagree / I rather agree / I fully agree / no answer
**I am concerned about what I consider novel mechanisms of action in COVID-19 vaccines.**
I do not agree at all / I rather do not agree / I neither agree nor disagree / I rather agree / I fully agree / no answer
**The development and approval of COVID-19 vaccines are moving too fast for me.**
I do not agree at all / I rather do not agree / I neither agree nor disagree / I rather agree / I fully agree / no answer
**I generally trust the COVID-19 vaccines.**
I do not agree at all / I rather do not agree / I neither agree nor disagree / I rather agree / I fully agree / no answer
**I believe the pharmaceutical industry cares more about its profit motives than safety of COVID-19 vaccines.**
I do not agree at all / I rather do not agree / I neither agree nor disagree / I rather agree / I fully agree / no answer
**I trust the European regulatory authorities of COVID-19 vaccines used in Germany.**
I do not agree at all / I rather do not agree / I neither agree nor disagree / I rather agree / I fully agree / no answer
**I generally trust in the German health care politics.**
I do not agree at all / I rather do not agree / I neither agree nor disagree / I rather agree / I fully agree / no answer
**I generally trust in the medical opinion of physicians.**
I do not agree at all / I rather do not agree / I neither agree nor disagree / I rather agree / I fully agree / no answer
**I favor an introduction of compulsory vaccination for health care professionals.**
I do not agree at all / I rather do not agree / I neither agree nor disagree / I rather agree / I fully agree / no answer
**In case of approval by the European and German authorities, I would also be vaccinated with a vaccine not developed in America or Europe.**
I do not agree at all / I rather do not agree / I neither agree nor disagree / I rather agree / I fully agree / no answer
**If you are concerned about short-term adverse effects, what are they?**
Free text
**If you are concerned about long-term adverse effects, what are they?**
Free text
**Knowledge**
**Which of the statements about COVID-19 vaccines do you think are correct?**
**m-RNA vaccines stimulate the formation of protein structures that mimic parts of the viral envelope.**
true / false / I do not know / no answer
**m-RNA vaccines alter the genetic material**
true / false / I do not know / no answer
**COVID-19 vaccination can cause me to shed infectious viruses**

true / false / I do not know / no answer
**m-RNA is rapidly degraded in the body**
true / false / I do not know / no answer
**My primary care physician has given me ...**
advise to get vaccinated against COVID-19 / advise not to get vaccinated COVID-19 / We have not discussed the vaccination so far. / We have addressed the vaccination; my primary care physician has not made a recommendation for or against the vaccination. / no answer
**The majority of my colleagues have / would like to**
get vaccinated against COVID-19 / not get vaccinated against COVID-19 / not yet decided whether to get vaccinated / we have not discussed vaccination so far / answer
**The majority of my family / friends have or would like to**
get vaccinated against COVID-19 / not get vaccinated against COVID-19 / not yet decided whether to get vaccinated / we have not discussed vaccination so far / no answer
**I have given the majority of my patients (without contraindication) ...**
advise to get vaccinated against COVID-19 / advise not to get vaccinated COVID-19 / We have not discussed the vaccination so far / We have addressed the vaccination; I have not made a recommendation for or against the vaccination / no answer
**I have given my family / friends**
advise to get vaccinated against COVID-19 / advise not to get vaccinated COVID-19 / We have not discussed the vaccination so far / We have addressed the vaccination; I have not made a recommendation for or against the vaccination / no answer
**Because of the COVID-19 pandemic, I, or my family, have suffered economic losses.**
yes / no / I do not know / no answer
**From what media do you get your information on COVID-19 vaccination?**
Daily newspapers, weekly magazines (print) / Daily newspapers, weekly magazines (online) / Scientific journals (print) / Scientific journals (online) / Television / radio / social networks (e.g., Facebook) / messenger services (e.g., Twitter, Telegram) / Online video platforms (e.g., YouTube) / Websites / information portals of government health authorities (e.g., RKI, WHO) / Other -> Free text

Supplemental Table S3: Grouped Occupational Categories and Work Settings

Grouped Occupational Category	Occupational groups
Non-physician medical staff	Certified nurses<br>Other non-physician medical staff<br>Dentistry assisting personnel
Physicians	Residents<br>Physician with specialist / personnel responsibility<br>Dentist
Administration / Science / Other	Administration / Science<br>Other
Students	Students of Human Medicine<br>Students of Dentistry

Grouped Work Setting Category	Work Settings
Hospital	Maximal-care hospital / university hospital<br>Hospital of other care levels
Medical practice, medical care center, vaccination centers, outpatient medical services	Medical practice / medical care center
out-of-hospital care and rescue service, specialized outpatient palliative care (non-physician)	Rescue service<br>Nursing home / retirement home<br>Outpatient nursing service

Supplemental Table S4: Category construction experienced side effects of prior vaccinations

Upper category	Fine category	Mentions
Allergic reaction	Allergy	Allergy, Body swollen, anaphylactic shock
Vaccination reaction	Fever	prolonged high fever after flu vaccination, Febrile seizure as child, After influenza vaccination fever up to 40 degrees
Vaccination reaction	Local reaction	pronounced local pain, Swelling + functional limitation upper arm
Vaccination reaction	Flu	Strong flu-like infection after flu vaccination, Flu, flu-like infection
Vaccination reaction	Dizziness	Dizziness
Vaccination reaction	Chills	Chills
Vaccination reaction	Fatigue	Exhaustion, chronic fatigue, tiredness
Vaccination reaction	Pain	Arthralgia, Bone pain
Vaccination reaction	Nausea	Nausea, vomiting
Skin damage	Skin	Exanthema, Inflammation of the puncture site and extensive inflammation of the subcutaneous fatty tissue
Cardiopulmonary side effects	Respiration	Asthma, Dyspnea, Bronchitis
Cardiopulmonary side effects	Circulation	Collapse, After flu vaccination inpatient treatment for cardiac complaints, Palpitations
Neurological Side effects	Neurological side effects	Seizures, nerval damage, Paresthesia at the vaccination arm, Sensory disturbances, visual disturbances
Other immune reaction	Immune side effects	Immunodeficiency, Tetanus vaccination -> exaggerated immune reaction, Idiopathic thrombocytopenic purpura
Limitation in daily life after vaccination	Ability to perform	Loss of condition, Lying in bed for 2 weeks, 2 weeks sick leave after flu vaccination
Other	other	Overvaccination, Mercury extremely high levels
Unclear / non-codable	Non-codable information	Non-codable information
	Other person	*The side effects involved another person. Side effects were assigned to the appropriate upper category.*

Supplemental Table S5: Category construction of feared short-term side effects

Upper category	Fine category	Mentions
Allergic reaction	Allergy	Allergic shock, anaphylactic shock
Vaccination reaction	Fever	Fever
Vaccination reaction	Chills	Chills
Vaccination reaction	Fatigue	listlessness, tiredness, exhaustion, sluggishness
Vaccination reaction	Malaise	Malaise
Vaccination reaction	Illness symptoms	General symptoms of illness, feeling ill, symptoms of illness, "that I feel very bad" General symptoms
Vaccination reaction	Infection	Infection-like symptoms
Vaccination reaction	Cold	Cold
Vaccination reaction	Flu	Flu-like symptoms
Vaccination reaction	Weakness	Feeling of weakness, lack of strength
Vaccination reaction	Dizziness	Dizziness
Vaccination reaction	local reaction /arm pain	Pain in the arm/at the injection site, inflammation at the injection site, paralysis of the arm, numbness of the vaccination arm, pain in the delta, "Local reaction"
Vaccination reaction	Aches	Pain general, joint pain, arthralgia, muscle pain, myalgia, muscle soreness
Vaccination reaction	Limb pain	Limb pain
Vaccination reaction	Headache	Headache
Vaccination reaction	Tiredness	Fatigue, tiredness, sleepiness
Limitation in daily life after vaccination	Drop in performance	Functional limitations, "not being fit", reduced performance, poor performance, restrictions in everyday life, not being able to regain my previous good health, low resilience
Limitation in daily life after vaccination	Sick leave	Loss of ability to work or perform; sick leave, inability to work
Allergic reaction	Shock	Shock
Other	Blood count	Thrombocytopenia
Neurological side effects	Neurological side effects	Neurologic NW, paresis, nerve damage, tremor, paralysis, convulsion, insipidity, migraine, facial paresis, Guillain-Barré syndrome
Cardiopulmonary side effects	circulatory	Circulatory problems, weakness, dysregulation, shock, presyncope, systemic reaction, cardiac symptoms, palpitations, cardinal side effects, fainting, collapse, pulmonary embolism
Cardiopulmonary side effects	Cardiopulmonary	Cardiopulmonary

Cardiopulmonary side effects	Respiratory	Breathing problems, lung pain, bronchitis, pneumonia, ventilation, coughing
COVID infection	COVID-Infection	Get infection/corona, attenuated infection with outbreak, side effects making more susceptible to covid 19
Vaccination reaction	Vaccination reaction	Vaccination reaction
Other immune reaction	Autoimmune, immune system	Immune reaction, autoimmune, immunodeficiency, cytokine storm, lymph node swelling, excessive antibody formation Autoimmune allergic reaction, temporary susceptibility to other infections, autoimmune allergic reaction
Death	Death	Death
Infertility	infertility	infertility
Liver/kidney damage	organ damage	Liver damage, organ damage, dialysis
Other immune reaction	pre-existing condition	negative influence of an existing immunological pre-existing disease, worsening of my underlying disease
Vaccination reaction	Nausea	Nausea
Vaccination reaction	Vomiting	Vomiting
Vaccination reaction	gastrointestinal	gastrointestinal symptoms, diarrhea
Skin damage	Skin	Skin side effects, tissue necrosis, erythema, Lyell's syndrome.
Other	Other (symptoms/diseases)	b-symptoms, health damage, psychogenic reaction, arthritis, disharmonies of the body, concentration disorders, myelitis, joint complaints, intolerance, blindness
Other	Other	Economic losses, Positive PCR tests, so quarantine, insufficient protection
	Non-codable	Non-codable information

Supplemental Table S6: Category construction feared long-term side effects

**Upper category**	**Fine category**	**Mentions**
Autoimmune / immune	Autoimmune<br>Autoimmune diseases	Autoimmune reaction/processes, autoimmune diathesis, activation of immune system against own proteins, cross-reaction against body's own proteins, possible relapse trigger autoimmune disease, Hashimoto's, rheumatism, lupus
Autoimmune / immune	Immune system<br>Concerning the immune system	Immune system in general, immune deficiency, immune response, damage to immune system, loss of own defenses, cytokine storm, immune modulation, higher susceptibility to other pathogens, protracted inflammation

Allergic reaction	Allergies	Allergy
Other	general	all possible, all possibly occurring
Limitation in daily life after vaccination	Limitations<br><br>Physical and health restrictions and impairments	...that my normal life is affected; Vitality limitations; Physically serious limitations; Unclear. Limitations of any kind; Other health limitations, physical limitations
Limitation in daily life after vaccination	Mental	psychological impairment, whether it changes the nature/psyche; psychological damage
Neurological side effects	Neurological<br><br>Neurological and cognitive side effects	Neurodegenerative diseases, dementia, nerve damage, facial paresis, neuritis, taste/smell disorders, migraine, MS, neuro-muscular complications, cognitive side effects, intellectual ability impairment, brain damage, epilepsy, concentration impairment, mental damage, memory impairment, cerebral side effects, Kreuzfeld-Jakob, myelitis, meningitis
Unpredictable side effects	Testing<br><br>Lack of studies/evidence due to novelty.	no studies on possible long-term side effects,
Questionable effectiveness	Effect<br><br>Concerns related to the efficacy of the vaccine	Long-term immunity possible?; Resistance developments in the virus; There is no long-term effect at all; Lack of effect, getting Corona; Whether revaccination is necessary; Vaccination failure
Unpredictable side effects	Unpredictable<br><br>Unknown/unpredictable/uncertain NW	Unforeseeable damage; So far unknown vaccination consequences; Unknown risks of the new vaccines; Diseases that cannot be estimated now, because they have not occurred yet; I don't know!; Uncertainty; Diffuse fears of not being able to estimate the situation, Unspecific
Other	Additives<br><br>NW caused by substances contained in the vaccine	General late effects, due to ingredients which are actually unnecessary; unexpected reaction to nanoparticles; exposure to the additives
Cell / Genome	cell	Previously completely unknown mechanisms in cell biology due to newly generated proteins; cell death; cell changes that can no longer be controlled; cell damage; cell death and replacement by connective tissue
Other	Metabolism<br><br>Metabolic problems, endocrinological side effects	Metabolic problems, endocrinological NW, influence on hormone balance

Chronic Damage	Chronic<br><br>Chronic and permanent damage or disease	chronic inflammatory disease; chronic complaints/diseases/cold symptoms; permanent/permanent damage ("of any kind"); development of chronic diseases. (rheumatism, multiple sclerosis.); development of other (organ.) diseases; long-term damage; severe diseases; general damage; rheumatic complaints; vaccination damage; severe vaccination consequences; health damage in general; kidney diseases
Limitation in daily life after vaccination	Pain	Pain
Other	Blood<br><br>Side effects affecting the blood count	Blood clot formation, vascular damage, thrombosis
Cardiopulmonary side effects	Cardiovascular	Cardiovascular, myocarditis, systemic diseases
Limitation in daily life after vaccination	Fatigue	Chronic Fatigue Syndrome, Fatigue, Fatigue Syndrome, Permanent Fatigue, Fatigue Syndrome, Fatigue, Sluggishness, Long Covid Syndrome
Fertility / Reproduction / Child	Fertility<br><br>Concerning the reproduction and pregnancy	Fertility disorders, impotence, infertility, influence on desire to have children, effects on subsequent pregnancies, limitations in family planning, damage to germ cells, teratogenicity, pregnancy problems
Fertility / Reproduction / Child	Child<br><br>Effects on later/unborn children	that it can have embryotoxic effects, damage to my child (I am still breastfeeding), malformations
Cancer	Cancer	Cancer, (increased) cancer risk, tumor, carcinogenic side effects, oncogene, lymphoma/leukemia, carcinogenicity, malignancy
Cell / Genome	Genes<br><br>Genes<br><br>Concerning the genes/genetics/DNA	DNA, aberrant post-transcriptional gene regulation, such as microRNA, incorporation of vector vaccine DNA, changes in epigenetics, mutations, mutagenic properties, replication errors due to mRNA material in the body, protein synthesis, cellular reprogramming
Limitation in daily life after vaccination	Performance<br><br>Performance in everyday life	Limitation of performance, loss of power
Limitation in daily life after vaccination	Inability to work	Incapacity to work, loss of working hours

Neurological side effects	Guillain-Barré Syndrome	Guillain-Barré Syndrome
Cardiopulmonary side effects	Breathing	Lung problems, pulmonary symptoms, breathing difficulties, (lung volume); emboli; pulmonary dysfunction; pulmonary fibrosis; lung damage
Death	Death	premature end of life
Chronic Damage	Organs<br>Organ damage and diseases in general	organic diseases, changes in organs/tissues, damage to organ systems, damage to the organism
Kidney/Liver	Kidney<br>Kidney and liver damage	Renal insufficiency, kidney disease, kidney problems, glomerular nephritis
Neurological side effects	Narcolepsy	Narcolepsy
Autoimmune / immune	Pre-existing condition	Influence (worsening) of an existing previous disease, effect on existing (chronic) disease
Other	Other symptoms/diseases	Hair loss, disabilities, blindness, problems due to contamination of the vaccine, chronic nausea; severe disease progression; rare syndromes; muscular atrophies, myopathies; sepsis due to the needle stick; interference with the body; abscesses; physical alteration
Other	Other	Dependent on medication for the rest of my life; loss of workforce because they would not get vaccinated under any circumstances; that I would not get vaccinated in time
	Non-codable	*Non-codable answers*

Supplementary Table S7: Overall Results.

**Experienced serious adverse effects, requiring medical treatment**	**Overall (N=4459)**
No	4323
Yes	136
No answer	41
**Allergic reaction**	
No	4436 (99.5%)
Yes	23 (0.5%)
**Vaccination reaction**	
No	4407 (98.8%)
Yes	52 (1.2%)
**Skin damage**	
No	4430 (99.3%)
Yes	29 (0.7%)

**Cardiopulmonary side effects**	
No	4446 (99.7%)
Yes	13 (0.3%)
**Neurological Side effects**	
No	4446 (99.7%)
Yes	13 (0.3%)
**Other immune reaction**	
No	4452 (99.8%)
Yes	7 (0.2%)
**Limitation in daily life after vaccination**	
No	4457 (100.0%)
Yes	2 (0.0%)
**Other**	
No	4447 (99.7%)
Yes	12 (0.3%)
**Unclear / non-codable**	
No	4447 (99.7%)
Yes	12 (0.3%)
**Fear of Short-term side effects**	**Overall (N=4500)**
No	3826 (85.0%)
Yes	674 (15.0%)
Missing	0 (0.0%)
**Allergic reaction**	
No	4347 (96.6%)
Yes	153 (3.4%)
**Vaccination reaction**	
No	4024 (89.4%)
Yes	476 (10.6%)
**Limitation in daily life after vaccination**	
No	4444 (98.8%)
Yes	56 (1.2%)
**Neurological side effects**	
No	4453 (99.0%)
Yes	47 (1.0%)
**Cardiopulmonary side effects**	
No	4458 (99.1%)
Yes	42 (0.9%)
**COVID infection**	
No	4490 (99.8%)
Yes	10 (0.2%)
**Other immune reaction**	
No	4467 (99.3%)
Yes	33 (0.7%)
**Death**	
No	4491 (99.8%)
Yes	9 (0.2%)
**Infertility**	
No	4495 (99.9%)
Yes	5 (0.1%)
**Liver/kidney damage**	
No	4495 (99.9%)
Yes	5 (0.1%)
**Skin damage**	
No	4491 (99.8%)
Yes	9 (0.2%)
**Other**	
No	4465 (99.2%)
Yes	35 (0.8%)
**Non-codable**	
No	4490 (99.8%)
Yes	10 (0.2%)
**Feared long-term side effects**	***Overall (N=4500)***

No	3731 (82.9%)
Yes	769 (17.1%)
Missing	0 (0.0%)
**Autoimmune / immune**	
No	4291 (95.4%)
Yes	209 (4.6%)
**Allergic reaction**	
No	4478 (99.5%)
Yes	22 (0.5%)
**Other**	
No	4447 (98.8%)
Yes	53 (1.2%)
**Limitation in daily life after vaccination**	
No	4434 (98.5%)
Yes	66 (1.5%)
**Neurological side effects**	
No	4320 (96.0%)
Yes	180 (4.0%)
**Unpredictable side effects**	
No	4332 (96.3%)
Yes	168 (3.7%)
**Questionable effectiveness**	
No	4477 (99.5%)
Yes	23 (0.5%)
**Cell / Genome**	
No	4441 (98.7%)
Yes	59 (1.3%)
**Chronic Damage**	
No	4400 (97.8%)
Yes	100 (2.2%)
**Cardiopulmonary side effects**	
No	4454 (99.0%)
Yes	46 (1.0%)
**Fertility / Reproduction / Child**	
No	4355 (96.8%)
Yes	145 (3.2%)
**Cancer**	
No	4420 (98.2%)
Yes	80 (1.8%)
**Death**	
No	4491 (99.8%)
Yes	9 (0.2%)
**Kidney/Liver**	
No	4493 (99.8%)
Yes	7 (0.2%)
**Non-codable**	
No	4493 (99.8%)
Yes	7 (0.2%)

Feared short-term side effects and association to COVID-19 vaccination willingness

	**Hesitant (N=375)**	**Willing (N=4125)**	**Total (N=4500)**	**p value**
**Fears of Short-term side effects, any**	<0.001			
No	293 (78.1%)	3863 (93.6%)	4156 (92.4%)	
Yes	82 (21.9%)	262 (6.4%)	344 (7.6%)	
**Vaccination reaction**	0.953			
No	335 (89.3%)	3689 (89.4%)	4024 (89.4%)	
Yes	40 (10.7%)	436 (10.6%)	476 (10.6%)	
**Allergic reaction**	< 0.001			

No	343 (91.5%)	4004 (97.1%)	4347 (96.6%)	
Yes	32 (8.5%)	121 (2.9%)	153 (3.4%)	
**Limitation in daily life after vaccination**	0.194			
No	373 (99.5%)	4071 (98.7%)	4444 (98.8%)	
Yes	2 (0.5%)	54 (1.3%)	56 (1.2%)	
**Neurological side effects**	< 0.001			
No	359 (95.7%)	4094 (99.2%)	4453 (99.0%)	
Yes	16 (4.3%)	31 (0.8%)	47 (1.0%)	
**Cardiopulmonary side effects**	< 0.001			
No	361 (96.3%)	4097 (99.3%)	4458 (99.1%)	
Yes	14 (3.7%)	28 (0.7%)	42 (0.9%)	
**COVID infection**	< 0.001			
No	369 (98.4%)	4121 (99.9%)	4490 (99.8%)	
Yes	6 (1.6%)	4 (0.1%)	10 (0.2%)	
**Other immune reaction**	< 0.001			
No	360 (96.0%)	4107 (99.6%)	4467 (99.3%)	
Yes	15 (4.0%)	18 (0.4%)	33 (0.7%)	
**Death**	< 0.001			
No	369 (98.4%)	4122 (99.9%)	4491 (99.8%)	
Yes	6 (1.6%)	3 (0.1%)	9 (0.2%)	
**Infertility**	< 0.001			
No	372 (99.2%)	4123 (100.0%)	4495 (99.9%)	
Yes	3 (0.8%)	2 (0.0%)	5 (0.1%)	
**Liver/kidney damage**	0.010			
No	373 (99.5%)	4122 (99.9%)	4495 (99.9%)	
Yes	2 (0.5%)	3 (0.1%)	5 (0.1%)	
**Skin damage**	0.131			
No	373 (99.5%)	4118 (99.8%)	4491 (99.8%)	
Yes	2 (0.5%)	7 (0.2%)	9 (0.2%)	
**Other**	< 0.001			
No	358 (95.5%)	4107 (99.6%)	4465 (99.2%)	
Yes	17 (4.5%)	18 (0.4%)	35 (0.8%)	
**Non-codable**	< 0.001			
No	370 (98.7%)	4120 (99.9%)	4490 (99.8%)	
Yes	5 (1.3%)	5 (0.1%)	10 (0.2%)	
**Feared long-term side effects and association to COVID-19 vaccination willingness**				
**Feared long-term side effects, any**	< 0.001			
No	186 (49.6%)	3545 (85.9%)	580 (14.1%)	
Yes	189 (50.4%)	580 (14.1%)	769 (17.1%)	
**Autoimmune / immune**	< 0.001			
No	314 (83.7%)	3977 (96.4%)	4291 (95.4%)	
Yes	61 (16.3%)	148 (3.6%)	209 (4.6%)	
**Allergic reaction**	< 0.001			
No	363 (96.8%)	4115 (99.8%)	4478 (99.5%)	
Yes	12 (3.2%)	10 (0.2%)	22 (0.5%)	
**Other**	< 0.001			
No	358 (95.5%)	4089 (99.1%)	4447 (98.8%)	
Yes	17 (4.5%)	36 (0.9%)	53 (1.2%)	

**Limitation in daily life after vaccination**	0.117			
No	366 (97.6%)	4068 (98.6%)	4434 (98.5%)	
Yes	9 (2.4%)	57 (1.4%)	66 (1.5%)	
**Neurological side effects**	< 0.001			
No	334 (89.1%)	3986 (96.6%)	4320 (96.0%)	
Yes	41 (10.9%)	139 (3.4%)	180 (4.0%)	
**Unpredictable side effects**	< 0.001			
No	335 (89.3%)	3997 (96.9%)	4332 (96.3%)	
Yes	40 (10.7%)	128 (3.1%)	168 (3.7%)	
**Questionable effectiveness**	< 0.001			
No	365 (97.3%)	4112 (99.7%)	4477 (99.5%)	
Yes	10 (2.7%)	13 (0.3%)	23 (0.5%)	
**Cell / Genome**	< 0.001			
No	353 (94.1%)	4088 (99.1%)	4441 (98.7%)	
Yes	22 (5.9%)	37 (0.9%)	59 (1.3%)	
**Chronic Damage**	< 0.001			
No	350 (93.3%)	4050 (98.2%)	4400 (97.8%)	
Yes	25 (6.7%)	75 (1.8%)	100 (2.2%)	
**Cardiopulmonary side effects**	0.052			
No	367 (97.9%)	4087 (99.1%)	4454 (99.0%)	
Yes	8 (2.1%)	38 (0.9%)	46 (1.0%)	
**Fertility / Reproduction / Child**	< 0.001			
No	321 (85.6%)	4034 (97.8%)	4355 (96.8%)	
Yes	54 (14.4%)	91 (2.2%)	145 (3.2%)	
**Cancer**	< 0.001			
No	356 (94.9%)	4064 (98.5%)	4420 (98.2%)	
Yes	19 (5.1%)	61 (1.5%)	80 (1.8%)	
**Death**	< 0.001			
No	367 (97.9%)	4124 (100.0%)	4491 (99.8%)	
Yes	8 (2.1%)	1 (0.0%)	9 (0.2%)	
**Kidney/Liver**	< 0.001			
No	374 (99.7%)	4119 (99.9%)	4493 (99.8%)	
Yes	1 (0.3%)	6 (0.1%)	7 (0.2%)	
**Non-codable**	0.016			
No	372 (99.2%)	4121 (99.9%)	4493 (99.8%)	
Yes	3 (0.8%)	4 (0.1%)	7 (0.2%)	

Feared short-term side effects and association to profession

	Non-physician medical staff (N=859	Physicians (N=1841)	Administration / Science / Other (N=414)	Students (N=1365)	NA (N=21)	Total (N=4500)	p value
**Short-term side effect, any**							
No	796 (92.7%)	1672 (90.8%)	383 (92.5%)	1287 (94.3%)	18 (85.7%)	4156 (92.4%)	
Yes	63 (7.3%)	169 (9.2%)	31 (7.5%)	78 (5.7%)	3 (14.3%)	344 (7.6%)	
**Allergic reaction**							0.516

No	835 (97.2%)	1769 (96.1%)	399 (96.4%)	1324 (97.0%)	20 (95.2%)	4347 (96.6%)	
Yes	24 (2.8%)	72 (3.9%)	15 (3.6%)	41 (3.0%)	1 (4.8%)	153 (3.4%)	
**Vaccination reaction**							0.199
No	753 (87.7%)	1655 (89.9%)	363 (87.7%)	1234 (90.4%)	19 (90.5%)	4024 (89.4%)	
Yes	106 (12.3%)	186 (10.1%)	51 (12.3%)	131 (9.6%)	2 (9.5%)	476 (10.6%)	
**Limitation in daily life after vaccination**							0.024
No	854 (99.4%)	1808 (98.2%)	413 (99.8%)	1348 (98.8%)	21 (100.0%)	4444 (98.8%)	
Yes	5 (0.6%)	33 (1.8%)	1 (0.2%)	17 (1.2%)	0 (0.0%)	56 (1.2%)	
**Neurological side effects**							0.248
No	847 (98.6%)	1825 (99.1%)	408 (98.6%)	1353 (99.1%)	20 (95.2%)	4453 (99.0%)	
Yes	12 (1.4%)	16 (0.9%)	6 (1.4%)	12 (0.9%)	1 (4.8%)	47 (1.0%)	
**Cardiopulmonary side effects**							< 0.001
No	843 (98.1%)	1826 (99.2%)	407 (98.3%)	1362 (99.8%)	20 (95.2%)	4458 (99.1%)	
Yes	16 (1.9%)	15 (0.8%)	7 (1.7%)	3 (0.2%)	1 (4.8%)	42 (0.9%)	
**COVID infection**							< 0.001
No	856 (99.7%)	1835 (99.7%)	414 (100.0%)	1365 (100.0%)	20 (95.2%)	4490 (99.8%)	
Yes	3 (0.3%)	6 (0.3%)	0 (0.0%)	0 (0.0%)	1 (4.8%)	10 (0.2%)	
**Other immune reaction**							0.034
No	858 (99.9%)	1824 (99.1%)	410 (99.0%)	1355 (99.3%)	20 (95.2%)	4467 (99.3%)	
Yes	1 (0.1%)	17 (0.9%)	4 (1.0%)	10 (0.7%)	1 (4.8%)	33 (0.7%)	
**Death**							0.527
No	858 (99.9%)	1838 (99.8%)	414 (100.0%)	1360 (99.6%)	21 (100.0%)	4491 (99.8%)	
Yes	1 (0.1%)	3 (0.2%)	0 (0.0%)	5 (0.4%)	0 (0.0%)	9 (0.2%)	
**Infertility**							0.423
No	858 (99.9%)	1837 (99.8%)	414 (100.0%)	1365 (100.0%)	21 (100.0%)	4495 (99.9%)	
Yes	1 (0.1%)	4 (0.2%)	0 (0.0%)	0 (0.0%)	0 (0.0%)	5 (0.1%)	
**Liver/kidney damage**							0.505
No	857 (99.8%)	1839 (99.9%)	413 (99.8%)	1365 (100.0%)	21 (100.0%)	4495 (99.9%)	

Yes	2 (0.2%)	2 (0.1%)	1 (0.2%)	0 (0.0%)	0 (0.0%)	5 (0.1%)	
**Skin damage**							0.829
No	857 (99.8%)	1839 (99.9%)	413 (99.8%)	1361 (99.7%)	21 (100.0%)	4491 (99.8%)	
Yes	2 (0.2%)	2 (0.1%)	1 (0.2%)	4 (0.3%)	0 (0.0%)	9 (0.2%)	
**Other**							0.027
No	853 (99.3%)	1820 (98.9%)	412 (99.5%)	1360 (99.6%)	20 (95.2%)	4465 (99.2%)	
Yes	6 (0.7%)	21 (1.1%)	2 (0.5%)	5 (0.4%)	1 (4.8%)	35 (0.8%)	
**Non-codable**							< 0.001
No	858 (99.9%)	1834 (99.6%)	413 (99.8%)	1365 (100.0%)	20 (95.2%)	4490 (99.8%)	
Yes	1 (0.1%)	7 (0.4%)	1 (0.2%)	0 (0.0%)	1 (4.8%)	10 (0.2%)	
**Feared long-term side effects and association to profession**							
**Feared long-term side effects, any**							0.011
Yes	705 (82.1%)	1545 (83.9%)	321 (77.5%)	1145 (83.9%)	15 (71.4%)	3731 (82.9%)	
No	154 (17.9%)	296 (16.1%)	93 (22.5%)	220 (16.1%)	6 (28.6%)	769 (17.1%)	
**Autoimmune / immune**							0.412
No	829 (96.5%)	1753 (95.2%)	396 (95.7%)	1293 (94.7%)	20 (95.2%)	4291 (95.4%)	
Yes	30 (3.5%)	88 (4.8%)	18 (4.3%)	72 (5.3%)	1 (4.8%)	209 (4.6%)	
**Allergic reaction**							0.998
No	855 (99.5%)	1832 (99.5%)	412 (99.5%)	1358 (99.5%)	21 (100.0%)	4478 (99.5%)	
Yes	4 (0.5%)	9 (0.5%)	2 (0.5%)	7 (0.5%)	0 (0.0%)	22 (0.5%)	
**Other**							0.024
No	841 (97.9%)	1822 (99.0%)	409 (98.8%)	1355 (99.3%)	20 (95.2%)	4447 (98.8%)	
Yes	18 (2.1%)	19 (1.0%)	5 (1.2%)	10 (0.7%)	1 (4.8%)	53 (1.2%)	
**Limitation in daily life after vaccination**							0.019
No	842 (98.0%)	1819 (98.8%)	402 (97.1%)	1351 (99.0%)	20 (95.2%)	4434 (98.5%)	
Yes	17 (2.0%)	22 (1.2%)	12 (2.9%)	14 (1.0%)	1 (4.8%)	66 (1.5%)	
**Neurological side effects**							0.384
No	826 (96.2%)	1766 (95.9%)	391 (94.4%)	1316 (96.4%)	21 (100.0%)	4320 (96.0%)	

Yes	33 (3.8%)	75 (4.1%)	23 (5.6%)	49 (3.6%)	0 (0.0%)	180 (4.0%)	
**Unpredictable side effects**							0.024
No	834 (97.1%)	1776 (96.5%)	388 (93.7%)	1315 (96.3%)	19 (90.5%)	4332 (96.3%)	
Yes	25 (2.9%)	65 (3.5%)	26 (6.3%)	50 (3.7%)	2 (9.5%)	168 (3.7%)	
**Questionable effectiveness**							0.467
No	856 (99.7%)	1828 (99.3%)	411 (99.3%)	1361 (99.7%)	21 (100.0%)	4477 (99.5%)	
Yes	3 (0.3%)	13 (0.7%)	3 (0.7%)	4 (0.3%)	0 (0.0%)	23 (0.5%)	
**Cell / Genome**							0.110
No	851 (99.1%)	1811 (98.4%)	406 (98.1%)	1353 (99.1%)	20 (95.2%)	4441 (98.7%)	
Yes	8 (0.9%)	30 (1.6%)	8 (1.9%)	12 (0.9%)	1 (4.8%)	59 (1.3%)	
**Chronic Damage**							< 0.001
No	823 (95.8%)	1814 (98.5%)	403 (97.3%)	1339 (98.1%)	21 (100.0%)	4400 (97.8%)	
Yes	36 (4.2%)	27 (1.5%)	11 (2.7%)	26 (1.9%)	0 (0.0%)	100 (2.2%)	
**Cardiopulmonary side effects**							< 0.001
No	842 (98.0%)	1829 (99.3%)	403 (97.3%)	1359 (99.6%)	21 (100.0%)	4454 (99.0%)	
Yes	17 (2.0%)	12 (0.7%)	11 (2.7%)	6 (0.4%)	0 (0.0%)	46 (1.0%)	
**Fertility / Reproduction / Child**							< 0.001
No	825 (96.0%)	1810 (98.3%)	398 (96.1%)	1302 (95.4%)	20 (95.2%)	4355 (96.8%)	
Yes	34 (4.0%)	31 (1.7%)	16 (3.9%)	63 (4.6%)	1 (4.8%)	145 (3.2%)	
**Cancer**							0.727
No	842 (98.0%)	1808 (98.2%)	409 (98.8%)	1341 (98.2%)	20 (95.2%)	4420 (98.2%)	
Yes	17 (2.0%)	33 (1.8%)	5 (1.2%)	24 (1.8%)	1 (4.8%)	80 (1.8%)	
**Death**							0.811
No	857 (99.8%)	1836 (99.7%)	414 (100.0%)	1363 (99.9%)	21 (100.0%)	4491 (99.8%)	
Yes	2 (0.2%)	5 (0.3%)	0 (0.0%)	2 (0.1%)	0 (0.0%)	9 (0.2%)	
**Kidney/Liver**							0.014
No	854 (99.4%)	1840 (99.9%)	414 (100.0%)	1364 (99.9%)	21 (100.0%)	4493 (99.8%)	
Yes	5 (0.6%)	1 (0.1%)	0 (0.0%)	1 (0.1%)	0 (0.0%)	7 (0.2%)	
**Non-codable**							< 0.001

No	859 (100.0%)	1837 (99.8%)	414 (100.0%)	1363 (99.9%)	20 (95.2%)	4493 (99.8%)	
Yes	0 (0.0%)	4 (0.2%)	0 (0.0%)	2 (0.1%)	1 (4.8%)	7 (0.2%)	

Feared short-term side effects and ICU / non-ICU work setting

**Short-term side effect, any**	ICU work setting (N=1525)	Non-ICU work setting (N=1105)	Total: 2630	0.678
No	1028 (93.4%)	1425 (93.4%)	2453 (93.3%)	
Yes	77 (7.0%)	100 (6.6%)	177 (6.7%)	

Feared long-term side effects and ICU / non-ICU work setting

**Long-term side effect, any**	ICU work setting (N=1525)	Non-ICU work setting (N=1105)	Total	0.144
No	938 (84.9%)	1262 (82.8%)	2200 (83.7%)	
Yes	167 (15.1%)	263 (17.2%)	430 (16.3%)	

Feared short-term side effects and frequency of COVID-19 patient treatment

**Short-term side effect, any**	Never, N= 1729	<50% of workdays, N= 1815	>50% of workdays, N= 307	Every workday, N=171	Total, N= 4022	0.144
No	1612 (93.2%)	1670 (92.0%)	288 (93.8%)	152 (88.9%)	3722 (92.5%)	
Yes	117 (6.8%)	145 (8.0%)	19 (6.2%)	19 (11.1%)	300 (87.5%)	

Feared long-term side effects and frequency of COVID-19 patient treatment

**Long-term side effect, any**	Never, N= 1729	<50% of workdays, N= 1815	>50% of workdays, N= 307	Every workday, N=171	Total, N= 4022	0.592
No	1419 (82.1%)	1519 (83.7%)	258 (84.0%)	142 (142 (83.0%)	3338 (83.0%)	
Yes	310 (17.9%)	296 (16.3%)	49 (16.0%)	29 (17.0%)	684 (17.0%)	

Association to personal surrounding

Feared short-term side effects

Advise given by general practitioner

**Any feared short-term side effects**	**Advised for COVID-19 vaccination (N=518)**	**Advised against COVID-19 vaccination (N=41)**	**Did not talk about it (N=2220)**	**No advise given (N=75)**	**Total (N=2854)**
No	482 (93.1%)	33 (80.5%)	2042 (92.0%)	60 (80.0%)	2617 (91.7%)
Yes	36 (6.9%)	8 (19.5%)	178 (8.0%)	15 (20.0%)	237 (8.3%)

Majority of colleagues

**Any feared short-term side effects**	**Will get vaccinated (N=3602)**	**Will not get vaccinated (N=173)**	**Has not decided yet (N=393)**	**Did not talk about it (N=154)**	**Total (N=4322)**
No	3377 (93.8%)	130 (75.1%)	340 (86.5%)	143 (92.9%)	3990 (92.3%)
Yes	225 (6.2%)	43 (24.9%)	53 (13.5%)	11 (7.1%)	332 (7.7%)

Majority of family

Any feared short-term side effects	Will get vaccinated (N=3682)	Will not get vaccinated (N=251)	Has not decided yet (N=465)	Did not talk about it (N=67)	Total (N=4465)
No	3457 (93.9%)	194 (77.3%)	410 (88.2%)	63 (94.0%)	4124 (92.4%)
Yes	225 (6.1%)	57 (22.7%)	55 (11.8%)	4 (6.0%)	341 (7.6%)
**Feared long-term side effects**					
**Advise given by general practitioner**					
**Any feared short-term side effects**	**Advised for COVID-19 vaccination (N=518)**	**Advised against COVID-19 vaccination (N=41)**	**Did not talk about it (N=2220)**	**No advise given (N=75)**	**Total (N=2854)**
No	446 (86.1%)	24 (58.5%)	1829 (82.4%)	40 (53.3%)	2339 (82.0%)
Yes	72 (13.9%)	17 (41.5%)	391 (17.6%)	35 (46.7%)	515 (18.0%)
**Majority of colleagues**					
**Any feared short-term side effects**	**Will get vaccinated (N=3602)**	**Will not get vaccinated (N=173)**	**Has not decided yet (N=393)**	**Did not talk about it (N=154)**	**Total (N=4322)**
No	3081 (85.5%)	95 (54.9%)	285 (72.5%)	119 (77.3%)	3580 (82.8%)
Yes	521 (14.5%)	78 (45.1%)	108 (27.5%)	35 (22.7%)	742 (17.2%)
**Majority of family**					
**Any feared short-term side effects**	**Will get vaccinated (N=3682)**	**Will not get vaccinated (N=251)**	**Has not decided yet (N=465)**	**Did not talk about it (N=67)**	**Total (N=4465)**
No	3179 (86.3%)	126 (50.2%)	340 (73.1%)	56 (83.6%)	3701 (82.9%)
Yes	503 (13.7%)	125 (49.8%)	125 (26.9%)	11 (16.4%)	764 (17.1%)

1. Holzmann-Littig C, Braunisch MC, Kranke P, Popp M, Seeber C, Fichtner F, et al. COVID-19 Vaccination Acceptance and Hesitancy among Healthcare Workers in Germany. Vaccines. 2021;9(7):777.
